# Supplementary material for: Multiplex Recombinase Polymerase Amplification Assay for Simultaneous Detection of Treponema pallidum and Haemophilus ducreyi in Yaws-Like Lesions
Source: Trop Med Infect Dis. 2020 Oct 6;5(4):157. doi: 10.3390/tropicalmed5040157 (PMC7709673; doi:10.3390/tropicalmed5040157)
Supplement: Supplementary file 1 [file tropicalmed-05-00157-s001.pdf]

Tropical Medicine and Infectious Disease

Multiplex Recombinase Polymerase Amplification Assay for Simultaneous Detection of *Treponema pallidum* and *Haemophilus ducreyi* in Yaws-Like Lesions

Assay Optimization

Oligonucleotide Primer Design

PolA and *HhdA* genes of *T. pallidum* and *H. ducreyi*, respectively, were used as target sequences for RPA detection. RPA primers and probes were designed in accordance with the TwistDx manufacturer's recommendations (TwistDx Limited, United Kingdom). Three forward primers (FPs), 3 reverse primers (RPs) and one exo probe (P) each for *T. pallidum* and *H. ducreyi* (Table 1) were design by using the Molecular Evolution Genetics Analysis version 7 software (Mega7).

Table S1. Sequences for all Primers and Probes Used in the Assay Development.

Name	Sequence
TP_RPA-FP1	CAGTGTACATGTGCACGTAACACTAATGTCG
TP_RPA-FP2	GAGTGCACAGAACAGCATGGGGTATCTGCA
**TP _RPA-FP3**	**GTAACACTAATGTCGAGACTGAAAAGGAGTGC**
TP _RPA-RP1	GTGAGCGTCTCATCATTCCAAAGACGTCGA
**TP _RPA-RP2**	**GCATAACAAGTGTGAGCGTCTCATCATTC**
TP _RPA-RP3	GCATAACAAGTGTGAGCGTCTCATCATTC
**TP _RPA-Probe**	**GTATCTGCATCTGCTGTGCAGGATCCGGCATATGTCCAAGCTGTCATG**
Hd_RPA-FP1	GCGTCATTCCGCCATCGGCCATTACGCCTA
Hd _RPA-FP2	AGAAGTTAGCGTCATTCCGCCATCGGCCAT
**Hd _RPA-FP3**	**TACTCAGGCAACGGATACCCAACATGCA**
**Hd _RPA-RP1**	**TAAATGACCTGTAACAGCCTGATTTACCTC**
Hd_RPA-RP2	CTTGTTCAGTAATAACAGTGCCTGACAATGA
Hd _RPA-RP3	TAGCATCAATAGTGGTACCTTGTAA
**Hd_RPA-Probe**	**TACGCCTAAATCGTTAACTGCGGGATTAGGTATAGATGGCCATGGTAGT**

*Highlighted oligonucleotides were those selected for assay development and validation.

Primer Screening and Selection

The 3 forward primers and 3 reverse primers together with probe for each target were tested in combinations using 10^5 molecular standard DNA. The pairs with highest fluorescent intensity in the shortest time were selected and retested with 10^2 DNA copies. The pair with the best results for both target were selected (see Figure 1 and 2).

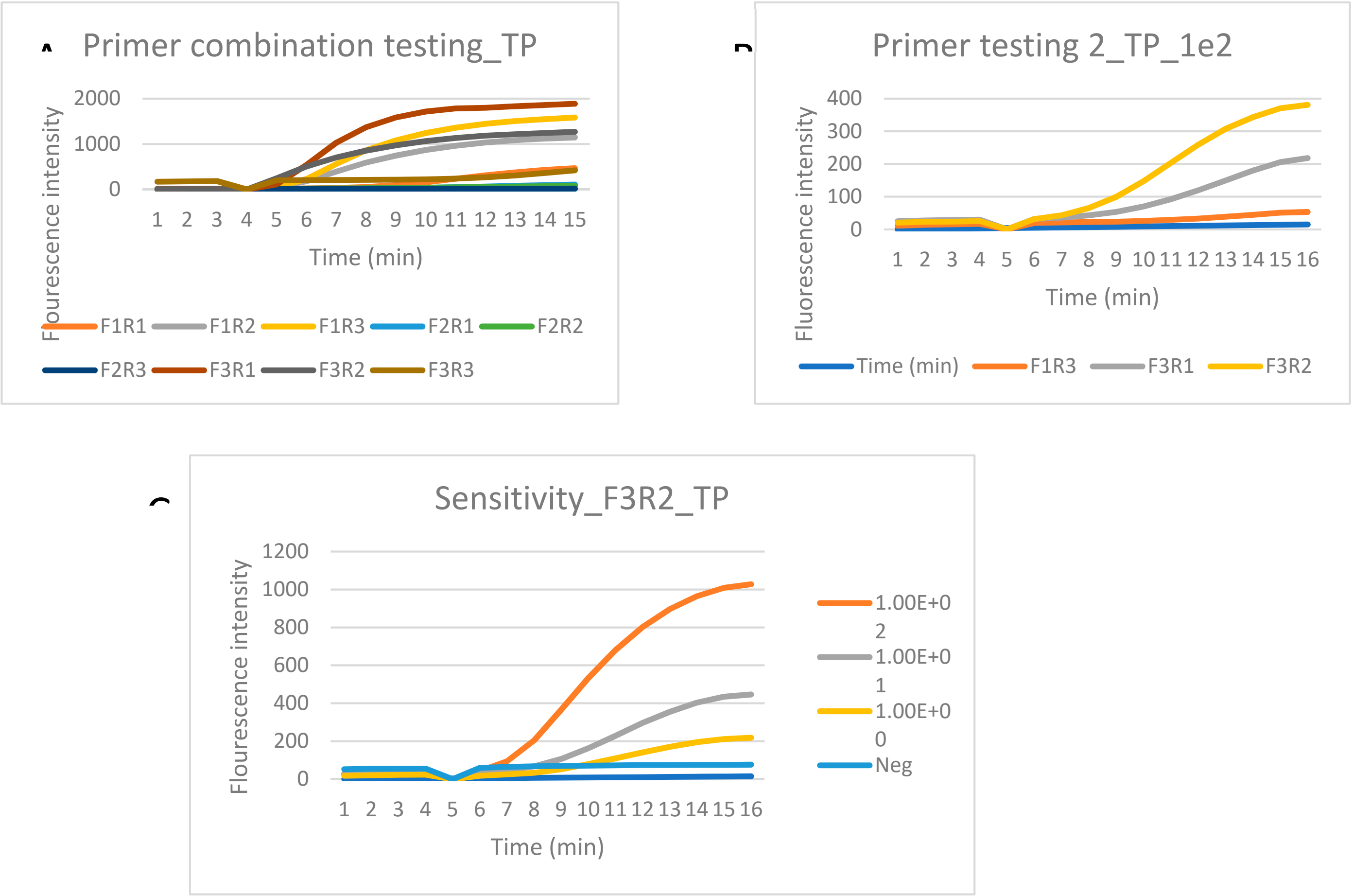

Figure S1. Primer combination testing for PolA of *T. pallidum*; (**A**) Each forward primer was paired and a reverse primer together with a probe (9 primer/probe pairs) and tested. (**B**) The 3 best performing pairs, F1R3, F3R1 and F3R2 were tested with a lower concentration of the standard DNA (**C**) The best of the 3 pairs, F3R2 was further tested with lower DNA copies to determine the lowest limit of detection.

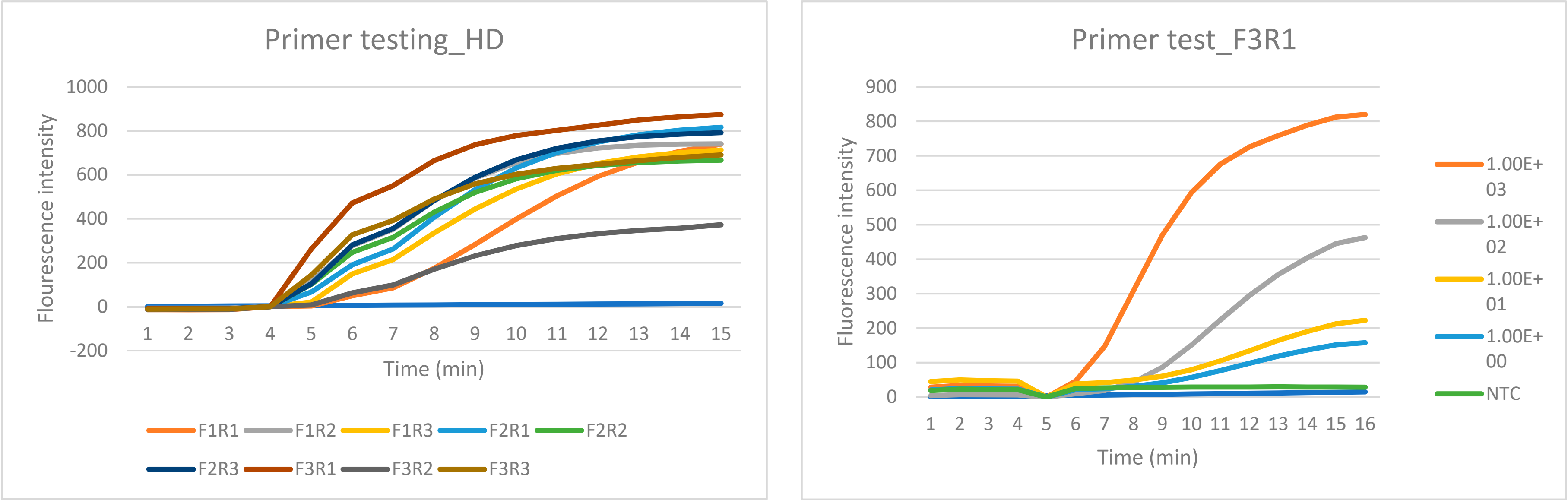

Figure S2. Primer combination testing for hhdA of *H. ducreyi*; (**A**) Each forward primer was paired and a reverse primer together with a probe (9 primer/probe pairs) and tested. (**B**) The best of the 3 pairs, F3R1 was further tested with lower DNA copies to determine the lowest limit of detection.

In silico Analysis of Primers and Probes

BLASTn analysis queries alignments were performed with the oligonucleotide primer and probe sequences of the TPHD-RPA duplex assay with publicly available nucleic acid sequences for *T. pallidum* and *H. ducreyi* in GenBank as of July, 2019 to demonstrate the predicted inclusivity TPHD duplex RPA assay. Results of BLAST analysis shown below;

**TP_RPA_FP3 (Forward Primer)**			
**Organisms**	**Sequence ID**	**Expect value**	**% homology**
Treponema pallidum subsp. pertenue strain CDC-1 chromosome	CP024750	7.00E-07	100
Treponema pallidum subsp. pertenue strain Sei Geringging K403 chromosome	CP024089	7.00E-07	100
Treponema pallidum subsp. pertenue strain Kampung Dalan K363 chromosome	CP024088	7.00E-07	100
Treponema pallidum subsp. pertenue strain LMNP-1 genome sequence	CP021113	7.00E-07	100
Treponema pallidum subsp. pertenue strain CDC 2575	CP020366	7.00E-07	100
Treponema pallidum subsp. pertenue strain Ghana-051	CP020365	7.00E-07	100
Treponema pallidum subsp. endemicum strain Iraq B chromosome	CP032303	7.00E-07	100
Treponema pallidum subsp. endemicum strain Iraq B DNA-directed DNA polymerase I (polA) gene, partial cds	KY120819	7.00E-07	100
Treponema pallidum subsp. endemicum isolate 11q/j DNA-directed DNA polymerase I (polA) gene, partial cds	KY120778	7.00E-07	100
Treponema pallidum subsp. endemicum str. Bosnia A genome	CP007548	7.00E-07	100
Treponema pallidum str. Fribourg-Blanc, complete genome	CP003902	7.00E-07	100
Treponema pallidum subsp. pertenue str. CDC2, complete genome	CP002375	7.00E-07	100
Treponema pallidum subsp. pertenue str. SamoaD, complete genome	CP002374	7.00E-07	100
Treponema pallidum strain UW-148B2 chromosome	CP045004	3.00E-05	100
Treponema pallidum strain UW-148B chromosome	CP045005	3.00E-05	100
Treponema pallidum PolA gene, partial cds	U57757	3.00E-05	100
Treponema pallidum subsp. pallidum strain X-4 chromosome, complete genome	CP040555	3.00E-05	100
Treponema pallidum subsp. pallidum strain Philadelphia 1 chromosome	CP035193	3.00E-05	100
Treponema pallidum subsp. pallidum strain Grady chromosome	CP035104	3.00E-05	100
Treponema pallidum subsp. pallidum strain Seattle Nichols genome	CP010422	3.00E-05	100
Treponema pallidum subsp. pallidum str. Nichols, complete genome	CP004010	3.00E-05	100
Treponema pallidum subsp. pallidum str. Nichols complete genome	AE000520	3.00E-05	100

TP_RPA_RP2 (Reverse Primer)			
**Organisms**	**Sequence ID**	**Expect value**	**% homology**
Treponema pallidum subsp. pallidum	CP040555	9.00E-06	100%
Treponema pallidum subsp. pertenue (yaws treponeme)	CP024750	9.00E-06	100%
Treponema pallidum subsp. endemicum	CP032303	9.00E-06	100%
Treponema pallidum subsp. endemicum str. Bosnia A	CP007548	9.00E-06	100%
Treponema pallidum subsp. pallidum str. Sea 81-4	CP003679	9.00E-06	100%
Treponema pallidum subsp. pallidum str. Nichols	CP004010	9.00E-06	100%
Treponema pallidum subsp. pallidum SS14	CP004011	9.00E-06	100%
Treponema pallidum str. Fribourg-Blanc	CP003902	9.00E-06	100%
Treponema pallidum subsp. pallidum str. Mexico A	CP003064	9.00E-06	100%
Treponema pallidum subsp. pallidum DAL-1	CP003115	9.00E-06	100%
Treponema pallidum subsp. pertenue str. Gauthier	CP002376	9.00E-06	100%
Treponema pallidum subsp. pertenue str. CDC2	CP002375	9.00E-06	100%
Treponema pallidum subsp. pertenue str. SamoaD	CP002374	9.00E-06	100%
Treponema paraluiscuniculi Cuniculi A	CP002103	9.00E-06	100%
Treponema pallidum subsp. pallidum str. Chicago	CP001752	9.00E-06	100%
Canis lupus familiaris (dog)	CP050574	8.4	65%
Neostethus bicornis	LR738563	8.4	93%
Sphaeramia orbicularis (orbiculate cardinalfish)	LR597473	8.4	62%
Cyanistes caeruleus (blue tit)	XM_023924328	8.4	65%
Ochrobactrum pituitosum	CP018782	8.4	65%
Mus musculus (house mouse)	AC130828	8.4	65%
Kosakonia sp. MUSA4	CP025697	33	62%
Schistosoma mansoni	HE601630	33	62%

TP_RPA_P1 (Probe)			
**Organisms**	**Sequence ID**	**Expect value**	**% homology**
Treponema pallidum subsp. pallidum	CP040555	5.00E-15	100%
Treponema pallidum subsp. pertenue (yaws treponeme)	CP024750	5.00E-15	100%
Treponema pallidum subsp. endemicum	CP032303	5.00E-15	100%
Treponema pallidum subsp. endemicum str. Bosnia A	CP007548	5.00E-15	100%
Treponema pallidum subsp. pallidum str. Sea 81-4	CP003679	5.00E-15	100%
Treponema pallidum subsp. pallidum str. Nichols	CP004010	5.00E-15	100%
Treponema pallidum subsp. pallidum SS14	CP004011	5.00E-15	100%
Treponema pallidum str. Fribourg-Blanc	CP003902	5.00E-15	100%
Treponema pallidum subsp. pallidum str. Mexico A	CP003064	5.00E-15	100%
Treponema pallidum subsp. pertenue str. Gauthier	CP002376	5.00E-15	100%
Treponema pallidum subsp. pertenue str. CDC2	CP002375	5.00E-15	100%
Treponema pallidum subsp. pertenue str. SamoaD	CP002374	5.00E-15	100%
Treponema paraluiscuniculi Cuniculi A	CP002103	5.00E-15	100%
Treponema pallidum subsp. pallidum str. Chicago	CP001752	5.00E-15	100%

Hd_RPA_FP3 (Forward Primer)			
**Organisms**	**Sequence ID**	**Expect value**	**% homology**
Haemophilus ducreyi	LR134364	3.00E-05	100%
[Haemophilus] ducreyi strain GHA3, complete genome	CP015431	3.00E-05	100%
[Haemophilus] ducreyi strain GHA1, complete genome	CP015429	3.00E-05	100%
[Haemophilus] ducreyi strain VAN3, complete genome	CP015426	3.00E-05	100%
[Haemophilus] ducreyi 35000HP	AE017143	3.00E-05	100%
Monodelphis domestica	XR_001622693	1.9	71%
Lotus japonicus	AP022631	29	67%
Legionella pneumophila subsp. pneumophila	CP016874	29	64%
Atlantibacter hermannii	CP049608	29	64%
Legionella pneumophila	CP048618	29	64%
Legionella pneumophila subsp. fraseri	CP017457	29	64%

Hd_RPA_RP1 (Forward Primer)

Organisms	Sequence ID	Expect value	% homology
[Haemophilus] ducreyi strain GHA5	CP015432	3.00E-06	100%
[Haemophilus] ducreyi strain GHA3	CP015431	3.00E-06	100%
[Haemophilus] ducreyi strain GHA2	CP015430	3.00E-06	100%
[Haemophilus] ducreyi strain VAN3	CP015426	3.00E-06	100%
[Haemophilus] ducreyi strain VAN2	CP015425	3.00E-06	100%
[Haemophilus] ducreyi strain 33921	CP011228	3.00E-06	100%
[Haemophilus] ducreyi strain NZV1	CP011227	3.00E-06	100%
[Haemophilus] ducreyi strain 82-029362	CP011222	3.00E-06	100%
[Haemophilus] ducreyi strain NZS4	CP011221	3.00E-06	100%
[Haemophilus] ducreyi 35000HP	AE017143	3.00E-06	100%
Pseudomonas aeruginosa	LR584237	0.6	64%
Gossypium raimondii	CP032557	2.4	64%
Pseudomonas phage PAN70	KJ959591	2.4	64%

Hd_RPA_P1 (Forward Primer)

Organisms	Sequence ID	Expect value	% homology
[Haemophilus] ducreyi strain GHA3, complete genome	CP015431	5.00E-15	100%
[Haemophilus] ducreyi strain GHA2, complete genome	CP015430	5.00E-15	100%
[Haemophilus] ducreyi strain VAN5, complete genome	CP015428	5.00E-15	100%
[Haemophilus] ducreyi strain NZS4 chromosome	CP011221	5.00E-15	100%
[Haemophilus] ducreyi strain NZS3 chromosome	CP011220	5.00E-15	100%
[Haemophilus] ducreyi 35000HP chromosome, complete genome	AE017143	5.00E-15	100%

Molecular Standard

Fragment DNA containing conserved regions for PolA gene of *T. pallidum* and HhdA gene of *H. ducreyi* was synthesized by GeneArt (Invitrogen, Darmstadt, Germany). We diluted the plasmid DNA in 10 mM Tris to adjusted concentration. The defined conserved regions used for the synthesis of the plasmid DNA are indicated below.

Treponema pallidum PolA gene, partial cds

GenBank: U57757.1

Nucleotide range:1836 -2135

GATTTTCGATCTGCTTTCAGGAAGCAGAGGCTTTTTATGTTCCTCTTATTGTTCCGGACG
TTTCTCTTCATACCGAGTCAACTCAGTGTACATGTGCACGTAACACTAATGTCGAGACTGAA
AAGGAGTGCACAGAACAGCATGGGGTATCTGCATCTGCTGTGCAGGATCCGGCATATGTCC
AAGCTGTCATGCACCAGCTTCGACGTCTTTGGAATGATGAGACGCTCACACTTGTTATGCAT
AATGGAAAGTTTGATTATCACGTTATGCATCGTGCAGGCGTTTTTGAGCACTGTG

Haemophilus ducreyi hemolytic cytotoxin HhdA and HhdB precursors (hhdA, hhdB) genes, complete cds

Sequence ID: U32175.1

Nucleotide range: 5031 – 5330 HhdA

GGTTAAAGCTGATAGCGCTAAATTTACTCAGGCAACGGATACCCAACATGCAAATGGT
ATAAAAGTTAGTGTAGAAGTGGGGGGAGAAGTTAGCGTCATTCCGCCATCGGCCATTACGC
CTAAATCGTTAACTGCGGGATTAGGTATAGATGGCCATGGTAGTGAGGTAAATCAGGCTGT
TACAGGTCATTTAAAGGGCGATAATGTGTCTATTGAAACTAAAAATGCTTTTTACAAGGTA
CCACTATTGATGCTAAAAATAAATCATTGTCAGGCACTGTTATTACTGAACAAGCACA

Analytical sensitivity and specificity

We have described how we determined the analytical sensitivity and limit of detection in the manuscript.

We generated analytical specificity data by in silico analysis. We then tested a panel of about 30 pathogens (table 2). In all cases the TPHD RPA assay tested negative, demonstrating high specificity for the assay.

Table 2. List of Bacteria and Parasite Species and Strains Used for Determining the Cross-Reactivity of the Duplex RPA Assay.

Name of Bacteria	Source
Nocardia ssp.	DSMZ ID: 43757
Enterococcus faecalis	DSMZ ID: 1103
Streptococcus agalactiae	DSMZ ID: 2134
Listeria monocytogenes	DSMZ ID: 15675
Staphylococcus aureus	DSMZ ID: 799
E. coli	DSMZ ID: 30083
Pseudomonas aeruginosa	DSMZ ID: 939
Clostridium perfringens	DSMZ ID: 756
Plasmodium falciparum	University of Ibadan, Nigeria
Leishmania major	
Leishmania donovani	American Type Culture Collection, Manassas, USA
Leishmania aethiopica	
Leishmania infantum	
Rickettsia africae	
Rickettsia rickettsia	BNITM Hamburg, Germany
Rickettsia conorii	
Rickettsia Helvetica	Bundeswehr, Muinch, Germany
Mycobacterium marinum	DSMZ ID: 44344
Mycabacterium smegmatis	DSMZ ID: 43756
Mycobacterium avium ssp. Avium	DSMZ ID: 44156
Mycobacterium avium ssp. silvaticum	DSMZ ID: 44175
Mycobacterium intracellulare	DSMZ ID: 43223
Mycobacterium fortuitum ssp. Fortuitum	DSMZ ID: 46621
Mycobacterium gordonae	DSMZ ID: 43213
Mycobacterium kansasii	DSMZ ID: 44162
Mycobacterium phlei	DSMZ ID: 43239
Mycobacterium avium paratuberculosis	ATCC 19638
Rhodococcus equi	ATCC 25729
Streptococcus uberis	DSMZ ID: 20569

© 2020 by the authors. Licensee MDPI, Basel, Switzerland. This article is an open access article distributed under the terms and conditions of the Creative Commons Attribution (CC BY) license (http://creativecommons.org/licenses/by/4.0/).
